# Novel *EGFR* ectodomain mutations associated with ligand-independent activation and cetuximab resistance in head and neck cancer

**DOI:** 10.1371/journal.pone.0229077

**Published:** 2020-02-18

**Authors:** Sindhu Nair, Hoa Q. Trummell, Rajani Rajbhandari, Nanda K. Thudi, Susan E. Nozell, Jason M. Warram, Christopher D. Willey, Eddy S. Yang, William J. Placzek, James A. Bonner, Markus Bredel

**Affiliations:** 1 Department of Radiation Oncology, University of Alabama at Birmingham, Birmingham, Alabama, United States of America; 2 Department of Otolaryngology, University of Alabama at Birmingham, Birmingham, Alabama, United States of America; 3 Department of Biochemistry and Molecular Genetics, University of Alabama at Birmingham, Birmingham, Alabama, United States of America; Marshall University, UNITED STATES

## Abstract

Epidermal growth factor receptor (EGFR) is a pro-tumorigenic receptor tyrosine kinase that facilitates growth for cancer cells that overexpress the receptor. Monoclonal anti-EGFR antibody Cetuximab (CTX) provides significant clinical benefit in patients with head and neck squamous cell carcinoma (HNSCC). Missense mutations in the ectodomain (ECD) of EGFR can be acquired under CTX treatment and mimic the effect of large deletions on spontaneous untethering and activation of the receptor. Little is known about the contribution of EGFR ECD mutations to EGFR activation and CTX resistance in HNSCC. We identified two concurrent non-synonymous missense mutations (G33S and N56K) mapping to domain I in or near the EGF binding pocket of the EGFR ECD in patient-derived HNSCC cells that were selected for CTX resistance through repeated exposure to the agent in an effort to mimic what may occur clinically. Structural modeling predicted that the G33S and N56K mutants would restrict adoption of a fully closed (tethered) and inactive EGFR conformation while not permitting association of EGFR with the EGF ligand or CTX. Binding studies confirmed that the mutant, untethered receptor displayed reduced affinity for both EGF and CTX but demonstrated sustained activation and presence at the cell surface with diminished internalization and sorting for endosomal degradation, leading to persistent downstream AKT signaling. Our results demonstrate that HNSCC cells can select for EGFR ECD mutations under CTX exposure that converge to trap the receptor in an open, ligand-independent, constitutively activated state. These mutants impede the receptor’s competence to bind CTX possibly explaining certain cases of CTX treatment-induced or de novo resistance to CTX.

## Introduction

Head and neck squamous cell carcinoma (HNSCC) is a biologically, phenotypically and clinically heterogeneous disease [1–3]. Epidermal growth factor (EGFR) is a paradigmatic receptor tyrosine kinase (RTK) that serves as a master conduit for many cell growth and differentiation pathways in this disease [4]. Moreover, inhibition of EGFR has become an important therapeutic target for these patients [5, 6].

EGFR is overexpressed in most and amplified and/or mutated in up to 15% of HNSCC [1]. Mutations involving the EGFR RTK domain usually lead to a constitutively active receptor [1]. Mutations in the ectodomain (ECD) of EGFR have been well-documented in other cancers [7–10]. Their contribution to HNSCC pathogenesis and therapy response has received little attention but could have therapeutic implications [7]. It has been demonstrated that EGFR ECD missense mutations can unexpectedly cause spontaneous receptor untethering that removes a restraint on RTK activation and that such mutants can be targeted by specific monoclonal antibodies (mAbs) [11].

The ECD of EGFR is composed of 4 discrete domains—two leucine-rich domains for ligand binding (I and III) and two cysteine-rich domains (II and IV) [12–14]. EGFR is activated by EGF-ligand binding to domains I and III that favors a conformational change of the ECD from a closed, self-inhibited ‘tethered’—locked by the molecular interaction between domain II and IV—to an open ‘untethered’ state [15]. This spatial rearrangement of the ECD exposes domains II and IV to bind to the corresponding domains of the adjacent receptor facilitating homo- or hetero-dimerization, auto-phosphorylation, and activation [12, 13, 15, 16]. Some evidence suggests that EGFR can preexist as an inactive dimer prior to ligand binding [17]. Upon ligand binding, the EGFR transmembrane domain rotates resulting in the reorientation of the intracellular RTK domain dimer from a symmetric inactive configuration to an asymmetric active configuration (‘rotational model’) [17]. This model helps explain how ECD missense mutations can potentially activate the receptor in the absence of EGF ligand without necessarily assuming that the mutations induce receptor dimerization [18]. This hypothesis is strengthened by recent evidence indicating that ECD missense mutations located at the domain I-II interface away from the self-inhibitory tether, can favor a third, untethered but compact intermediate EGFR conformation occurring transiently from the tethered-to-untethered transition [11]. This conformation originates from a rotation of ECD domain I—which binds EGF—and has been postulated to expose a cryptic, cancer-characteristic epitope in a similar way as does the constitutively active EGFRvIII mutant that lacks the ECD [11, 19]. These observations suggest that ECD missense mutations can have structural and functional consequences that are equivalent to large-spanning ECD deletion changes [11].

Current therapeutic strategies targeting the ECD of EGFR seek to competitively interfere with ligand binding at domains I and III [16, 20]. Cetuximab (CTX)—a therapeutic monoclonal antibody (mAb) [5, 21]—structurally inhibits the receptor by binding to domain III of EGFR’s tethered ECD, thereby sterically overlapping the ligand-binding site and stabilizing the receptor in the closed conformation [13, 16, 22, 23]. CTX provides significant clinical benefit in patients with HNSCC [5, 6]. However, treatment failure occurs and has been shown to correlate with biological elevation of EGFR expression [24], genetic or epigenetic alterations of the EGFR [25–28], or downstream targets [1, 3, 29, 30], impaired EGFR trafficking and degradation [31–33], or signaling through alternative RTKs [34]. A single case report has described CTX resistance in a HNCC patient as a result of an acquired CTX-binding site mutation in the EGFR ECD [35].

Herein, we characterize two novel EGFR ECD mutations that are concurrently selected for in patient-derived HNSCC cells while these cells were repeatedly exposed to CTX in an effort to mimic what may occur clinically. While the effect of small EGFR ECD missense mutations remain to be fully understood, we demonstrate that these mutations hinder EGF and CTX binding and are associated with ligand-independent activation of the receptor suggesting functional equivalence to large ECD deletion mutations. These findings have significance regarding methods of circumventing CTX resistance.

## Results

**Fig 1:**
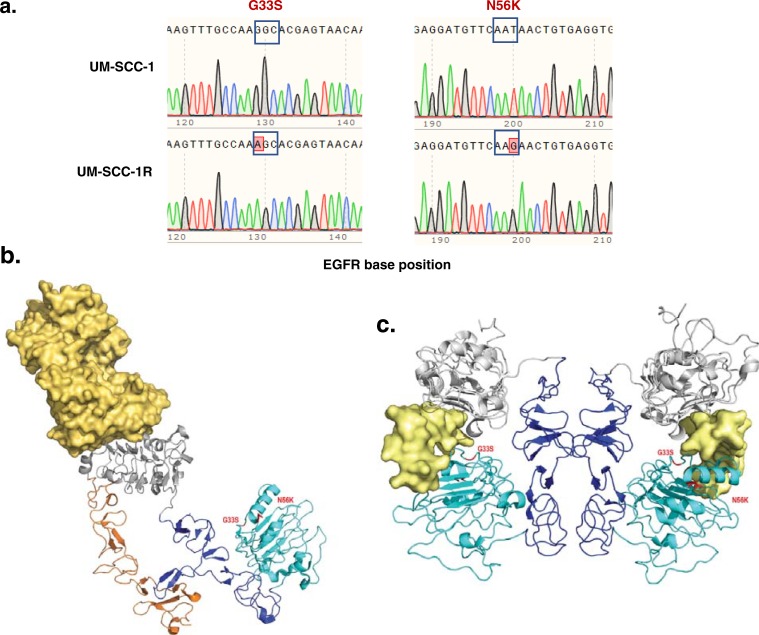
*EGFR* ectodomain mutants in the closed and open conformations. (**a**) EGFR G33S and N56K mutations identified in CTX-resistant UM-SCC-1R cells but not in parental UM-SCC-1 cells by Sanger sequencing. The locations of the two mutations (G33S and N56K) are highlighted in red in the closed (**b**) and open (**c**) conformations of EGFR. (**b**) The closed, tethered monomer confirmation (PDB:1yy9) is presented in complex with CTX (yellow space-filling) with ECD domain I (cyan), domain II (blue), domain III (grey), and domain IV (orange) shown in ribbons. (**c**) EGFR domains are colored as in (**b**) but are shown in the open, untethered/dimer complex confirmation bound to EGF (yellow space filling) and lack domain IV.

We selected patient-derived HNSCC cells (UM-SCC-1) for resistance by repeated, stepwise exposure to CTX in an attempt to recapitulate a clinical setting (termed UM-SCC-1R). We previously showed that the escape mechanism of these cells involved enhanced EGFR-induced downstream signaling without identifying a direct cause [21]. Recently, one study reported a G465R ECD mutation in EGFR affecting the CTX binding site on EGFR conducive to the development of resistance [35]. To date, a total of 19 mutations with respect to EGFR have been identified by The Cancer Genome Atlas (TCGA) HNSCC Project (https://portal.gdc.cancer.gov/projects/TCGA-HNSC) encompassing 17 missense mutations, one nonsense mutation, and one frameshift deletion. Amongst these, 14 mutations were specifically in the ECD. Therefore, we examined whether the resistance formation in our cells could be attributed to EGFR sequence changes. Subsequently, Sanger sequencing identified two novel EGFR ECD mutations (G33S, N56K, **Fig 1A**). The locations of the mutated residues are highlighted in crystal structures of the ECD in complex with CTX [16] and EGF [15] in **Fig 1B and 1C**.

It is known that CTX complexes with ECD domain III of EGFR in the closed confirmation and, by partially overlapping the ligand-binding site, prevents EGF binding [13, 16, 22, 23]. Therefore, we sought to determine the possible implications of the G33S and N56K ECD mutations on how they contribute to CTX resistance. Structural modeling shows the ECD-CTX complex (**Fig 1B**) with the ECD in its closed, tethered conformation while the EGF-bound structure (**Fig 1C**) highlights how rotation of domain II and III enables a dimeric form of the ECD in the open, untethered conformation and forms a pocket for EGF binding. In wild-type EGFR, domains I-III are arranged in a C shape and EGF is docked between domains I and III while a protruding beta-hairpin arm of each domain II holds the body of the other [36]. Structurally, G33S and N56K both mapped to ECD domain I [13]. In the closed conformation, G33S and N56K reside in a single shared pocket of the ECD with G33S situated at the end of the initial beta strand that makes contacts with domain II during formation of the closed conformation (**Fig 1B**). Following rotation, these mutants occupy distinct structural sites in the open conformation. G33S is positioned directly in the interface of domain I binding to EGF. N56K does not make direct contact with EGF in the open conformation, but rather sits at the C-terminal end of the first alpha helix in domain I that serves as the key interface between domain I and EGF (**Fig 1C**).

**Fig 2:**
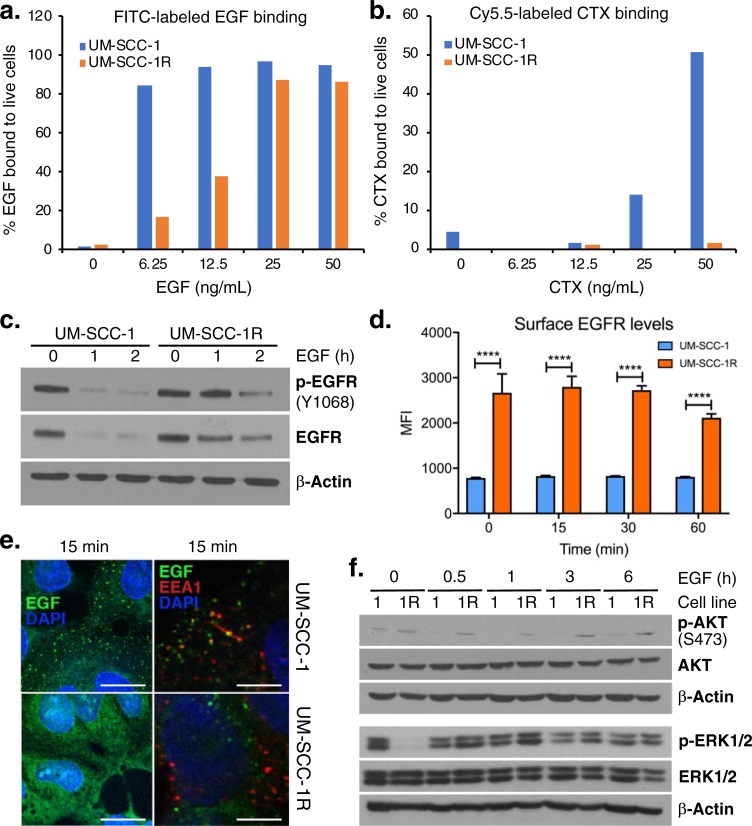
Effect of G33S and N56K mutants on EGF or CTX binding and EGFR activation and degradation. (**a-b**) FITC-labeled EGF and Cy5.5-labeled CTX in UM-SCC-1 vs.–SCC-1R (G33K-N56 mut) assessed by flow cytometry after 30 min of incubation with various concentrations of EGF or CTX. (**c**) Phospho- and total EGFR levels at indicated times of incubation with saturating EGF (60 ng/mL). (**d**) Surface levels of EGFR in cells stimulated with 60 ng/mL EGF in unpermeabilized/unfixed cells, by flow cytometry using a secondary goat anti-rabbit Alexa-Fluor 488 antibody. (**e**) Mapping of Alexa-Fluor 488-EGF conjugate shows internalization (green dotted lipid rafts) and co-localization with early endosome in UM-SCC-1 but not UM-SCC-1R. Scale bar represents 10 μM. (**f**) Consistently increased phospho-AKT but overall comparable (p)ERK1/2 levels in the mutant (1R = UM-SCC-1R) vs. parental (1 = UM-SCC-1) cells. Blots for (p)ERK1/2 were generated on a separate gel with its own β-actin loading control.

Given that our structural-based modeling mapped G33S and N56K into a shared EGF binding pocket, we assessed the competence of the mutant receptor to bind its own ligand. UM-SCC-1 and UM-SCC-1R cells were incubated with increasing concentrations of FITC-labelled EGF for 30 minutes, following which cells were analyzed by flow cytometry to assess ligand binding. We found that mutant UM-SCC-1R cells display diminished EGF binding affinity at low EGF concentrations (6.25–12.5 ng/mL) but that affinity increased and was almost similar to parental, non-mutant UM-SCC-1 cells at high EGF concentrations (25–50 mg/mL) (**Fig 2A**).

By contrast, UM-SCC-1 and UM-SCC-1R cells displayed notably distinct CTX binding dynamics at a broad range of concentrations of the mAb: while exposure to increasing concentrations (6.25–50 ng/mL) of CTX for 30 min led to increased binding of Cy5.5-labeled CTX in UM-SCC-1 cells, none of these concentrations reached meaningful binding in UM-SCC-1R cells (**Fig 2B**). Structural studies investigating the binding mechanisms of CTX have depicted the mAb as an antagonist by exclusively binding to domain III of the ECD of the tethered receptor, covering an epitope that partially overlaps the EGF binding site on that domain [16]. Therefore, G33S and N56K cannot directly explain the reduced CTX binding in the resistant cells. However, the CTX epitope—which we found to be not mutated (exon 12)—is fully exposed only in the transitional form of EGFR that occurs because the receptor changes from the inactive tethered conformation to an active untethered form [16]. Therefore, prolonged adoption of the extended conformation could indirectly impact the ability of CTX to bind the receptor. Stimulation of UM-SCC-1 and UM-SCC-1R cells with saturating doses (60 ng/mL) of EGF revealed sustained presence and activation of the receptor in UM-SCC-1R cells after 60 and 120 minutes (**Fig 2C**). Consistently, flow cytometry demonstrated prolonged high levels of the EGFR at the cell surface in response to saturating EGF doses in UM-SCC-1R compared to UM-SCC-1 cells, indicating impaired receptor internalization (**Fig 2D**).

Next, we examined this difference in receptor internalization with respect to intracellular trafficking of EGFR. Within minutes of activation, EGFR is typically internalized into endocytic vesicles and sorted into the endosomal machinery for recycling or degradation [37–47]. Receptor endocytosis is a spatiotemporally regulated process in which the internalized receptor is first shuttled to the early endosome followed by the late endosome and finally to the lysosome for degradation [48, 49]. We visualized and compared internalization of EGFR in UM-SCC-1 vs. UM-SCC-1R cells by stimulating cells with saturating EGF (60 ng/mL) conjugated to Alexa Fluor 488. We observed abundant internalization and dot-like clustering of EGF-EGFR complexes in raft-like domains at 15 min in UM-SCC-1 cells but hardly in UM-SCC-1R. Lipid rafts can sequester EGFR and reduce the number of receptors on the cell membrane [50]. Consistently, co-immunofluorescence confirmed greatly reduced co-localization with early endosome antigen 1 (EEA1) in UM-SCC-1R vs. UM-SCC-1 cells, implying diminished endosomal sorting and trafficking (**Fig 2E**). These findings are consistent with previous reports that suggest CTX-resistant cells have an impaired ability to efficiently sort EGFR for degradation leading to perpetual signaling [31–33]. EGFR phosphorylation and subsequent ubiquitination and degradation is an important determinant of response to cisplatin [51], a commonly used anticancer therapeutic in H&N cancers. Given the altered endosomal sorting dynamics of EGFR in UM-SCC-1R vs. UM-SCC-1 cells, we examined their sensitivity to cisplatin but did not note an appreciable difference in cell growth, proliferation, or colony formation assays (data not shown).

Finally, we probed downstream EGFR signaling as these events play an important role in the growth-promoting function of EGFR [20, 37]. We previously showed that UM-SCC-1R cells display increased phospho-serine 727 and total STAT3 –a key downstream target of EGFR–expression compared to UM-SCC-1 cells [21]. AKT and ERK1/2 are major EGFR-induced transforming pathway serine-threonine protein kinases. We found sustained phospho-AKT activation up to 6 hours following stimulation with saturating EGF in UM-SCC-1R but not in UM-SCC-1 but no meaningful difference in phospho-ERK1/2 levels (**Fig 2F**). A recent study investigating mechanisms of CTX resistance in HNSCC found that CTX therapy directly inhibited the activation of AKT in CAL33 HNSCC cells whereas CTX-resistant cells had constitutively activated AKT [31]. Inhibiting the PI3K/AKT pathway resulted in sensitivity towards CTX indicating that the AKT pathway has a direct role in CTX resistance [31]. Similarly, our CTX resistant cells also exhibit constitutive AKT activation suggesting that by selectively activating pro-survival pathways, CTX-resistant HNSCCs possibly ensure tumor growth and survival while also potentiating resistance in this scenario.

## Discussion

**Fig 3:**
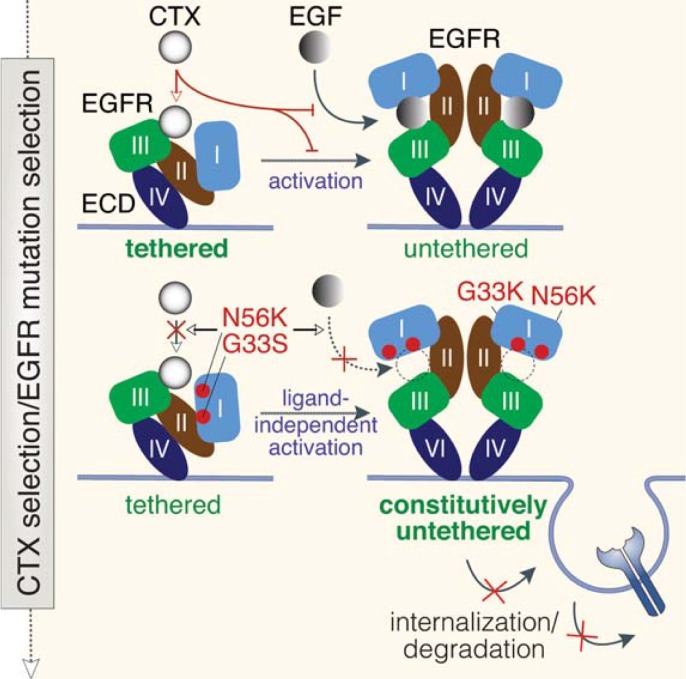
Model. Upon EGF binding, the ECD (contains domains I-IV) of EGFR switches from a closed, inactive (‘tethered’) state to an open, active (‘untethered’) state. Upon CTX selection (CTX interacts with domain III of tethered EGFR thereby preventing EGF binding), HNSCC cells acquire EGFR mutations (G33S, N56K) in domain I, which leads to ligand-independent activation and prevents receptor internalization/degradation. Mutant EGFR does not bind CTX since it is ‘trapped’ in the open confirmation, leading to CTX resistance.

A better understanding of acquired CTX resistance may lead to the development of new therapies to circumvent this resistance. Therefore, we explored CTX resistance in cells that were initially sensitive to CTX but formed CTX resistance after repeated exposure to CTX. In these studies, we identified novel missense mutations in the ECD of EGFR in patient-derived HNCC cells that render the receptor active independent of EGF ligand and resistant to CTX. Our data show that the G33S and N56K mutants impede EGFR internalization and sorting and sustain high levels of downstream signaling. Our finding of ligand-independent EGFR activation and concurrent CTX resistance as a consequence of mutations in or near the EGF binding pocket highlights the potentially profound impact and molecular mimicry small missense mutations can have on protein dynamics and function: restricting adoption of a fully closed, inactive EGFR conformation while not permitting association of EGFR with EGF; and, in parallel, restricting accessibility for domain III to interact with CTX, thereby leading to CTX resistance in our model (**Fig 3**).

Structural analysis of the ECD of EGFR has established compelling evidence that domains I, II, and III adopt a closed conformation in the absence of EGF and upon EGF binding undergo rotation of domains II and III to an extended and open conformation that exposes the EGF binding pocket [17]. This rotational model for EGF binding underscores the impact that protein dynamics have on function and further provides some insight into how the identified mutations may alter association with EGF and CTX. Importantly, the two mutations are each positioned to influence key interfaces necessary for adoption of the closed conformation with G33S and N56K located at or near the interface of domain I and EGF.

We were initially surprised that the CTX resistant cells showed sustained high levels of total and activated EGFR despite the low affinity for EGF, though these results mirror previous reports of ECD missense mutations—albeit in glioblastoma—which showed constitutive EGFR activity in the absence of EGF suggesting that ECD mutations can have tumorigenic receptor-activating potential [7, 52, 53]. Our subsequent structural analysis regarding the location of these mutations provided some insight into the observed phenotype. Specifically, the reduction in EGF affinity is likely a direct result of G33S and N56K impacting the receptor’s affinity towards EGF as these are both positioned in or near the EGF binding pocket. Likewise, the CTX resistance of the mutants can be possibly explained by the constitutive presence of EGFR in an untethered state, which perturbs the normal closed conformation of the ECD and thus limits availability of domain III to associate with CTX thereby decreasing affinity. Thus, the identified mutations could have the impact of both restricting the adoption of a fully closed and inactive EGFR conformation while not allowing association of EGFR with EGF. This restriction may be expected to alter the accessibility for domain III to interact with CTX, thereby leading to the reduction in CTX association observed in our assay. While our data establish that continuous exposure to an anti-EGFR agent can select for EGFR ectodomain mutations that are associated with low affinity to that agent or EGFR, full validation of the significance of these mutants will require combinations of site-directed mutagenesis and wild-type EGFR knockout experiments in additional patient-derived H&N cancer cell lines.

Our results add to an emerging body of evidence suggesting that EGFR ECD missense mutations can cause spontaneous EGFR untethering that promotes activation of the RTK [11]. Missense mutations located at the domain I-II interface away from the self-inhibitory tether, have been shown to increase ECD flexibility to an open conformation by removing an ECD fragment that acts as steric hinderance to prevent RTK activation [11]. Such heterogenous ECD mutants can present opportunities for molecular targeting and are for example responsive to cancer-specific mAbs [7, 11, 54–57]. Moreover, first-in-class anti-EGFR mixtures of recombinant, human-mouse chimeric mAbs have also demonstrated some initial promise to overcome CTX resistance mediated by EGFR ECD mutations [54, 57].

## Materials & methods

### Head and neck squamous cell carcinoma cells

Patient-derived HNSCC cells, UM-SCC-1, were acquired from Dr. Thomas Carey at the University of Michigan. Additional details, including genotyping, origin and unique cell identity, have been reported in [58]. Cells were grown in Dulbecco’s Modified Eagle Medium (MT-10-090-CV, Gibco) supplemented with 10% fetal bovine serum (Sigma) and 5% Pen-strep (Corning) and treated with 5 μg/ml of CTX (Eli Lilly & Co) for six months to create CTX-resistant cells denoted as UM-SCC-1R as previously described [21]. Acquisition of resistance was observed by absence of cell death in HNSCC cells and confirmation of a viable population of resistant cells by periodic cell counts using Coulter cell counter. Cells have been sporadically tested for pathogens by Charles River Research Animal Diagnostic Services, and all the results were negative.

### Genomic DNA and mRNA extraction and analysis

Genomic DNA was isolated from cells using GenElute Mammalian Genomic DNA Miniprep Kit (Sigma G1N70-1KT). Standard Sanger Sequencing with BigDye v3.1 (AppliedBiosystems) chemistry was performed and the samples were run on an ABI 3730xl Genetic Analyzer. Total RNA was extracted from cells using Trizol. To assess mRNA expression levels, 1 μg of total RNA was reverse transcribed and analyzed by quantitative polymerase chain reaction (PCR). Reactions for each sample were performed in triplicate using a PCR protocol (95°C activation for 10 min followed by 40 cycles of 95°C for 15 sec and 60°C for 1 min) in an ABI StepOnePlus Detection System (Applied Biosystems). Quantitative RT-PCR (qRT-PCR) was performed via TaqMan Assay (Applied Biosystems).

### Structural modeling

Mammalian EGFR is composed of four extracellular domains, named I, II, III, and IV, that alter their conformation in response to ligand binding. To model our point mutations onto EGFR in the CTX- and EGF-bound conformations, we downloaded Protein Databank (PDB) coordinates for the x-ray crystal structures of CTX- and EGF-bound forms of EGFR, 1yy9 and 1ivo, respectively. The CTX-EGFR complex (1yy9) includes coordinates for all four extracellular domains while the EGF-EGFR complex (1ivo) shows the dimer structure of EGFR with domains I, II, and III. We separated the respective domains into independent elements using PyMOL molecular visualization software (https://pymol.org) and identified the location of our point mutation in each model. The impact of specific mutations was assessed through visual analysis of space filling models of the native sidechains in the original structures and comparing this with their respective side-chain mutations. Domains that contain a point mutation were depicted as cartoons, with the location of the point mutation highlighted in a different color while domains or proteins that did not contain mutations were shown in space filling models.

### Immunoblotting

Cells were grown to 70% confluency and then serum starved overnight. Whole cell lysate was collected in RIPA buffer supplemented with protease inhibitor cocktail (100nM PMSF, 100mM sodium orthovanadate, 2.5 mg/ml aprotinin, 2.5 mg/ml leupeptin, 5nM Sodium Fluoride). Protein was resolved through SDS-PAGE under denaturing conditions, transferred to polyvinylidene difluoride (PVDF) membrane, and blocked in 5% non-fat milk in TBS-T. Subsequent incubation with the indicated antibody was done overnight at 4°C. Incubation with HRP-conjugated secondary in TBS containing 5% nonfat milk was performed for 1 hour and protein was detected using ECL chemiluminescence methods (Pierce ThermoScientific, Grand Island, NY).

### Reagents and antibodies

Reagents and antibodies were obtained from the following sources: EGF from Fisher Scientific, Alexa Fluor 594 conjugated secondary (A11032,Thermo Scientific), β-Actin (3700, Cell Signaling), total EGFR (4267, Cell Signaling), phospho-EGFR (3777, Cell Signaling), Akt (9272, Cell Signaling), phospho-Akt (4058, Cell Signaling), phospho-ERK (9101, Cell Signaling), total ERK (9102, Cell Signaling), anti-mouse and anti-rabbit secondary IgG-conjugated horse radish peroxidase (7074; 7076, Cell Signaling).

### Flow cytometry

Cells were treated with varying concentrations of FITC-labeled EGF (Thermofisher) and Cy 5.5-labeled CTX respectively for 30 minutes at 37°C. Cells were collected and analyzed by BD LSR II flow cytometer for the percentage bound fraction of labeled EGF ligand or labeled CTX.

### Internalization and co-localization studies

To assess EGF internalization, cells were pre-cooled to 4°C for 30 minutes and then treated with 25 ng/ml of EGF conjugated to Alexa Fluor 488 (E13345, ThermoFisher). After incubation at 4°C for 90 minutes, cells were transferred to 37°C for appropriate time points, washed in ice cold PBS and fixed in 4% paraformaldehyde. For co-immunostaining studies, cells were treated as described above, followed by permeabilization in 0.1% Tween in PBS (PBS-T) for 10 minutes at room temperature followed by blocking in 5% bovine serum albumin in PBS at room temperature. Overnight incubation at 4°C in primary antibody against EEA1 (3288, Cell Signaling) at 1:100 dilution was carried out after which cells were washed three times in PBS-T (0.1% Tween in PBS) and incubated in anti-rabbit secondary antibody (Alexa Flour 594) at 1: 200 dilution for 1 hour at room temperature. Cells were washed three times in PBS-T and mounted in Prolong anti-fade diamond mountant with DAPI (P36962, ThermoFisher) and imaged using a Nikon A1R confocal microscope.

### Statistics

Studies have been designed to incorporate multiple treatment conditions, often applying a full factorial design, for experiments that are continuous in nature. For data summary purposes, means and standard deviations were calculated within each experimental condition and plots were examined to diagnose extreme outliers. Formal analysis, where only one experimental factor was varied, used one-way ANOVA to evaluate global differences across groups; two-way ANOVA was applied when multiple experimental factors were varied. Because of the number of statistical hypothesis tests being evaluated, multiple comparisons adjustments were not performed; rather nominal p-values <0.05 along with consistent interpretations of mechanisms over the series of experiments were used to avoid false positive conclusions. For pairwise comparisons, triplicates in each experimental condition afford 80% power at two-sided Type I error to detect differences in continuous outcomes of approximately 3 standard deviations using a t-test. All other studies used continuous readouts of binding characteristics and cell-specific marker expression to evaluate the impact of EGFR ECD mutations.

## Supporting information

S1 Raw images Original blot images. (DOCX)
