# Supplementary material for: Novel EGFR ectodomain mutations associated with ligand-independent activation and cetuximab resistance in head and neck cancer
Source: PLoS One. 2020 Feb 18;15(2):e0229077. doi: 10.1371/journal.pone.0229077 (PMC7028269; doi:10.1371/journal.pone.0229077)
Supplement: S1 Raw images — (DOCX) [file pone.0229077.s001.docx]

ORIGINAL BLOT IMAGES

Figure 2C - **pEGFR , β-Actin**


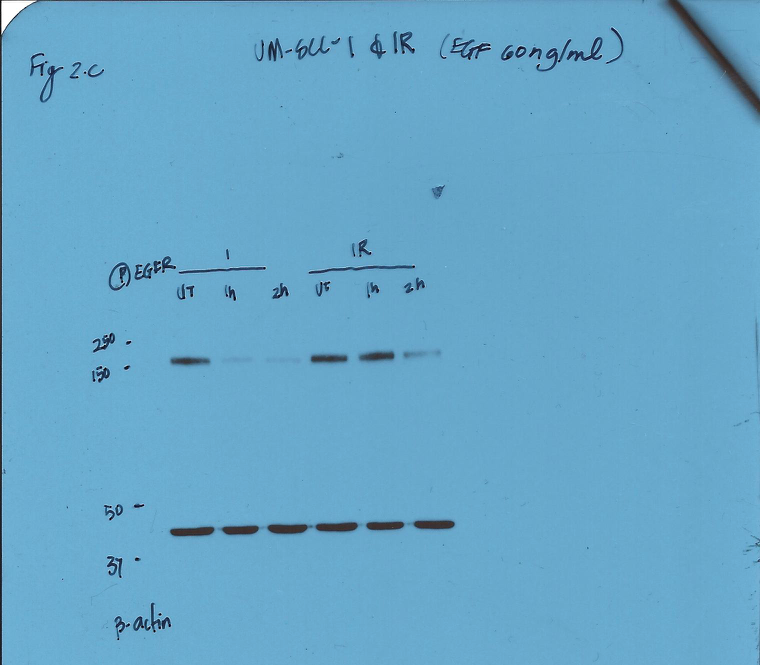


Figure 2C - **EGFR, β-Actin**


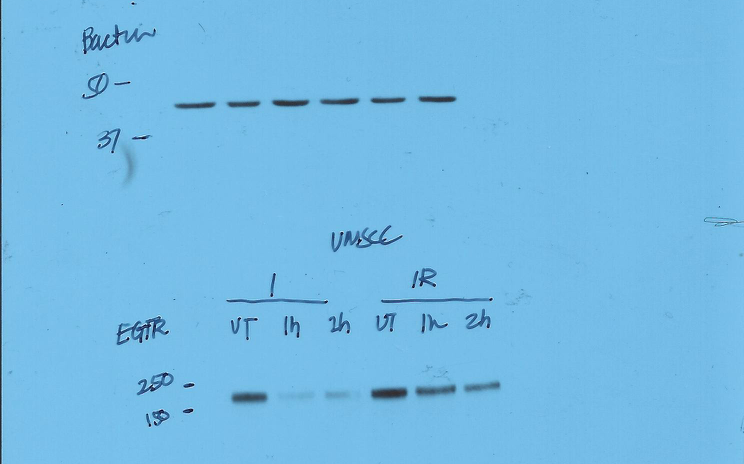


Figure 2F - **pAKT, AKT and β-Actin**

Figure 2F **- p-ERK1/2**

Figure 2F **- ERK 1/2**

Figure 2F - **β-Actin [corresponding to (p)ERK1/2]**

**
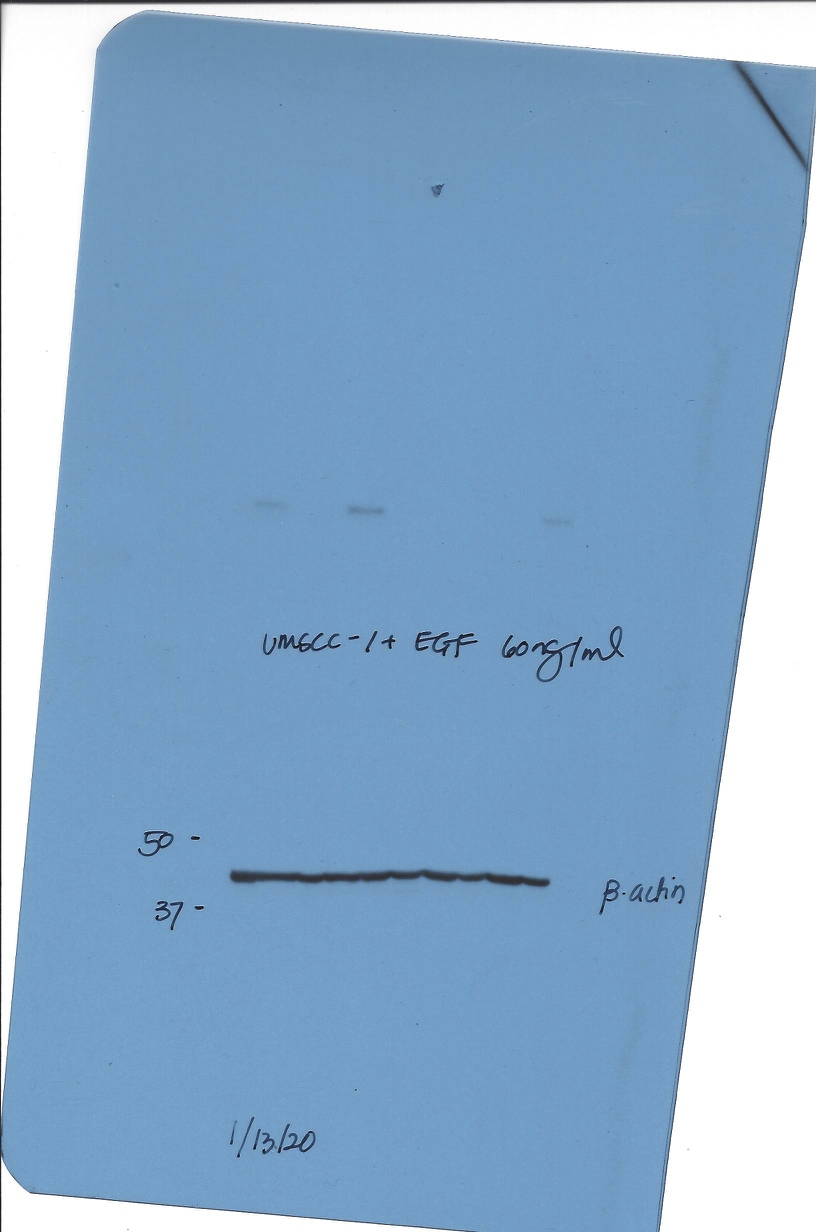
**
